# Locus of Control Moderates the Effect of Segregation on Everyday Functioning of Black Older Adults in the ACTIVE Study

**DOI:** 10.1093/geroni/igaf122.2162

**Published:** 2025-12-31

**Authors:** Abbey Hamlin, Alexandra Weigand, Ashley Chikkala, Michael Marsiske, Shannon Sisco, Wassim Tarraf, Kelsey Thomas, Alexandra Clark

**Affiliations:** University of Texas at Austin, Austin, Texas, United States; University of California, San Francisco, San Francisco, California, United States; University of Texas at Austin, Austin, Texas, United States; University of Florida, Gainesville, Florida, United States; Michael E. DeBakey VA Medical Center, Houston, Texas, United States; Wayne State University, Detroit, Michigan, United States; University of California, San Diego, La Jolla, California, United States; University of Texas at Austin, Austin, Texas, United States

## Abstract

Residential segregation has been linked to accelerated declines in everyday functioning for Black older adults, and there is a need to better understand how individual-level resilience factors may help mitigate the impact of structural inequities. Locus of control (LOC) is the extent to which individuals believe life outcomes are determined by internal or external forces. Higher internal LOC has been associated with better cognition, whereas higher external LOC has been linked to poorer outcomes. Given LOC may be an important psychological mechanism to consider in the face of adversity, the present study examined whether LOC moderates the effects of segregation on everyday functioning. Data came from 682 Black older adults from the ACTIVE study who completed a self-report measure of LOC. Baseline residential addresses were used to characterize segregation as measured by tract level dissimilarity (uneven spatial distribution of racial groups) and interaction (likelihood of contact between racial groups). Everyday functioning was measured at baseline, post-intervention, 1-, 2-, 3-, 5-, and 10-year follow-ups. Latent growth curve models, adjusted for demographic, health, and study characteristics, revealed a significant LOC x dissimilarity effect (B=-.45, p=.002); lower external LOC was associated with higher levels of everyday functioning for those living in more segregated versus less segregated tracts. No significant LOC x interaction effect on everyday functioning was observed (Bs ≤ .25, ps ≥ .43). Results suggest that LOC is an important psychological factor to capitalize on while we continue to address unjust and unfair policies that have led to inequalities.

